# Redox Homeostasis and Oxidative Stress in Schizophrenia: Glutathione–NMDA–Neuroimmune Convergence and a Hypothesis-Generating Iron–Lipid Redox Extension

**DOI:** 10.3390/ijms27167105

**Published:** 2026-08-08

**Authors:** Dušan Mihajlo Spasić, Snežana Spasić, Aleksandra Nikolić-Kokić, Čedo Miljević

**Affiliations:** 1 Department of Pharmacology, Clinical Pharmacology and Toxicology, Faculty of Medicine, University of Belgrade, 11000 Belgrade, Serbia; 2 Department of Chemistry, Institute of Chemistry, Technology and Metallurgy, National Institute of the Republic of Serbia, University of Belgrade, 11000 Belgrade, Serbia; svujin@chem.bg.ac.rs; 3 Department of Physiology, Institute for Biological Research “Siniša Stanković”—National Institute of the Republic of Serbia, University of Belgrade, 11000 Belgrade, Serbia; 4 Institute of Mental Health, Faculty of Medicine, University of Belgrade, 11000 Belgrade, Serbia; cedo.miljevic35@gmail.com

**Keywords:** schizophrenia, redox dysregulation, oxidative stress, glutathione, N-methyl-D-aspartate receptor, parvalbumin interneurons, neuroinflammation, mitochondria, iron–lipid redox regulation, ferroptosis-related mechanisms

## Abstract

Schizophrenia is a heterogeneous neurodevelopmental disorder in which genetic liability, developmental exposures, illness stage, treatment, and metabolic or inflammatory comorbidity may shape redox biology. This narrative review evaluates the human and mechanistic evidence for impaired redox adaptation as a convergence mechanism linking glutathione (GSH) regulation, N-methyl-D-aspartate receptor hypofunction, parvalbumin-interneuron and perineuronal-net vulnerability, mitochondrial–glial dysfunction, and neuroimmune signalling. The findings do not support a uniform oxidative abnormality across all patients or compartments: the peripheral biomarkers are heterogeneous, the group-level brain GSH magnetic resonance spectroscopy findings are largely null, and treatment-related changes vary by marker and illness phase. Beyond the established GSH–NMDA–redox–immune models, we integrate iron–lipid redox regulation as a conditional, hypothesis-generating extension and apply a deliberately conservative evidence hierarchy. Human studies more often report a lower peripheral iron and reduced or redistributed brain iron than a uniform iron excess; the plasma signals for predominantly intracellular proteins remain analytically unvalidated, and ferroptotic neuronal death has not been demonstrated. Longitudinal, sex-aware, multi-compartment, and challenge-based studies are needed to define meaningful redox subgroups; no redox- or ferroptosis-related biomarker is currently validated to guide treatment selection.

## 1. Introduction

Schizophrenia is a clinically and biologically heterogeneous syndrome characterised by positive symptoms, negative symptoms, cognitive impairment, altered social functioning, and diverse longitudinal outcomes. Contemporary models place its origins within a prolonged neurodevelopmental trajectory in which polygenic liability interacts with prenatal, perinatal, adolescent, and adult exposures before overt illness emerges [1,2].

Redox biology is well-positioned to connect these levels because the reactive-species production, antioxidant capacity, mitochondrial bioenergetics, immune activation, glutamatergic signalling, and neuronal plasticity form an adaptive network. Redox dysregulation is not proposed as a single sufficient cause of schizophrenia. Rather, it is a context-dependent convergence mechanism through which multiple risks may influence the circuit maturation, stress resilience, and recovery [3,4,5,6].

The central thesis is narrower than a claim of generalised oxidative excess. Depending on the illness stage and biological compartment, an altered redox profile may reflect the pre-existing vulnerability, incomplete inducibility of protective programmes, compensation, acute decompensation, medication exposure, or progression-associated injury. Blood, brain spectroscopy, post-mortem tissue, patient-derived cells, animal models, and *in vitro* experiments answer different questions and are not interchangeable sources of causal evidence.

Steullet et al. conceptualised redox dysregulation, neuroinflammation, and NMDAR hypofunction as a central hub, while Dwir et al. emphasised redox–immune signalling and therapeutic potential. Lv and Luo subsequently provided a dedicated review of canonical ferroptosis mechanisms and proposed therapeutic avenues in schizophrenia [4,5,7]. Against this background, the present review’s contribution is primarily evidentiary: it embeds iron–lipid redox regulation as a conditional, hypothesis-generating extension within the broader redox–NMDA–immune model and deliberately grades each link by evidence type, biological compartment, illness stage, analytical validity, null findings, and competing explanations. Within this integrated perspective, ferroptosis is discussed as a potential mechanism arising from disturbed iron–lipid redox regulation, and the current evidence is considered in relation to the broader spectrum of redox disturbances implicated in schizophrenia.

The review therefore evaluates the replicated human findings, negative or inconsistent evidence, functional genetic and omics data, post-mortem observations, patient-derived cellular models, and preclinical mechanisms. Therapeutic and biomarker implications are considered only after separating these evidentiary levels.

## 2. Methods

This narrative review was developed through targeted searches of PubMed/MEDLINE and backward and forward citation screening up to 22 July 2026. To improve reproducibility, Supplementary Table S1 provides the grouped search strings, date limits, and the number of references retained in the final synthesis for each domain. The literature search followed a purposive, iterative approach; consequently, raw database hit counts were not prospectively logged. Priority was given to systematic reviews and meta-analyses, larger or longitudinal human studies, post-mortem and multimodal investigations, functional genetic studies, and mechanistic reports needed to test the proposed network.

Reporting and structure were self-audited against the six SANRA domains: justification of the review’s importance, statement of aims, description of the literature search, referencing, presentation of evidence levels, and presentation of relevant endpoint data [8]. Evidence was classified descriptively as follows: (i) replicated or quantitatively synthesised human evidence; (ii) single-cohort human associations, including cross-sectional peripheral biomarkers; (iii) post-mortem or patient-derived cellular observations; (iv) preclinical mechanistic evidence; or (v) hypothesis-generating inference. Evidence tiers are assigned at the level of each principal finding in Table 1.

Narrative certainty descriptors (moderate, low, and very low) are based on GRADE terminology, but are applied descriptively within the narrative evidence framework used in this review [39]. These descriptors are interpretive summaries rather than formal GRADE ratings because study selection and extraction were not duplicated and no study-level risk-of-bias instrument was applied. Article selection was purposive and organised around the review questions rather than a prospectively registered systematic-review protocol. The search was limited to one bibliographic database, supplemented by citation screening; relevant records indexed elsewhere may therefore have been missed. These features limit claims of comprehensiveness and increase susceptibility to selection and publication bias.

## 3. Brain Redox Homeostasis: GSH, Parallel Defences, and Redox-Sensitive Cellular Targets

Reactive oxygen species (ROS) and reactive nitrogen species (RNS) are not intrinsically pathological. At low, spatially constrained concentrations, they participate in receptor modulation, transcriptional control, synaptic plasticity, stress adaptation, and immune defence. Oxidative distress develops when radical, peroxide, and reactive-nitrogen production exceeds the antioxidant, repair, and metabolic capacity, causing lipid, protein, nucleic-acid, and bioenergetic injury [40,41].

The brain is vulnerable because it consumes substantial oxygen, depends heavily on mitochondrial ATP production, contains abundant oxidisable polyunsaturated membrane lipids, and handles redox-active metals. Mitochondrial electron leakage, NADPH oxidases, activated microglia, and dopamine auto-oxidation or enzymatic metabolism are context-dependent sources of reactive species [42,43,44,45].

GSH is the principal non-enzymatic intracellular redox buffer emphasised in this review. It supplies reducing equivalents to glutathione peroxidases, maintains protein thiols, contributes to hydrogen-peroxide and lipid-hydroperoxide detoxification, and links antioxidant defence to glutamate, cysteine, and glycine metabolism. Synaptic NMDAR activity can regulate the transcription of the GSH system, whereas GSH depletion can alter the redox-sensitive receptor function, calcium handling, mitochondrial resilience, and Nrf2 or NF-kappaB signalling [3,46].

This focus should not be interpreted as implying that the GSH–GPX4 axis is the only antioxidant or anti-ferroptotic system. The thioredoxin–peroxiredoxin network, catalase, superoxide dismutases, NADPH-generating pathways, membrane-repair processes, and CoQ- or tetrahydrobiopterin-dependent defences provide parallel and partially compensatory protection. Their relevance becomes particularly important when interpreting a normal GSH measure or considering ferroptosis-related mechanisms.

Several schizophrenia-relevant cellular systems have high redox demands. Fast-spiking parvalbumin-positive interneurons support gamma-band synchrony; perineuronal nets stabilise their maturation and firing properties; oligodendrocyte-lineage cells support myelination and long-range connectivity; and coordinated neuron-astrocyte programmes sustain glutamate recycling, metabolic buffering, and antioxidant support [24,47,48].

**Figure 1:**
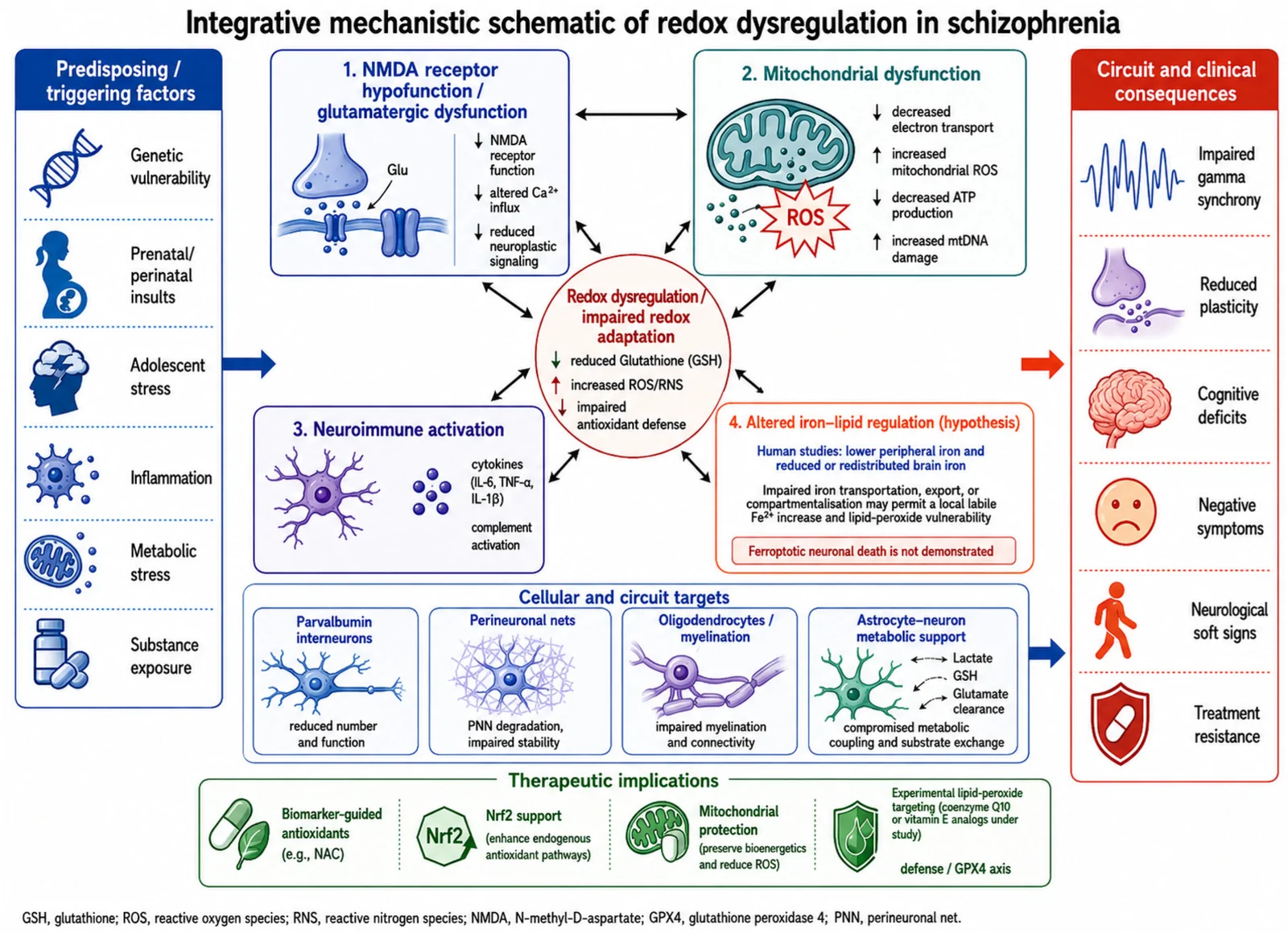
Integrative mechanistic schematic of redox dysregulation in schizophrenia. Genetic and environmental vulnerability may interact with impaired redox adaptation, NMDAR dysfunction, mitochondrial–glial stress, neuroimmune signalling, and altered iron–lipid redox regulation, with downstream effects on redox-sensitive cells, circuits, and clinical phenotypes. The arrows represent proposed reciprocal interactions rather than equivalent levels of evidence or a linear causal pathway. Panel 4 distinguishes bulk iron from a hypothetical local labile pool; the evidentiary boundaries for this Tier v extension and for ferroptosis-related terminology are defined in Section 9. Treatment resistance is shown only as a tentative research endpoint, and the schematic does not imply that all mechanisms are present in every patient. Abbreviations: GSH, glutathione; GPX4, glutathione peroxidase 4; NAC, N-acetylcysteine; NMDAR, N-methyl-D-aspartate receptor; NSS, neurological soft signs; PNN, perineuronal net; PV, parvalbumin; ROS/RNS, reactive oxygen or nitrogen species.

Figure 1 summarises the broad conceptual convergence framework. The evidentiary boundaries for the iron–lipid redox extension is defined in Section 9.

## 4. Human Evidence: Peripheral, Central, Longitudinal, and Inconsistent Findings

Peripheral studies provide the largest human evidence base, but their direction and interpretation vary with the illness stage, specimen, medication, smoking, diet, inflammation, and metabolic status. In a meta-analysis of 44 studies, the total antioxidant status was reduced in first-episode psychosis but increased after the antipsychotic treatment of acute psychosis; red-blood-cell catalase and plasma nitrite behaved more like state markers, whereas red-blood-cell SOD was reduced across the acute relapse, first episode, and stable outpatient states [9]. These findings support the stage sensitivity rather than one invariant biochemical signature.

Peripheral blood is not a single analytical compartment. Mature erythrocytes are anucleate and lack mitochondria; erythrocyte SOD, catalase, or GPX activity can characterise red-cell redox status but cannot directly assay mitochondrial bioenergetics, Nrf2/SIRT1 transcriptional inducibility, or leukocyte inflammatory signalling. Plasma and serum reflect extracellular chemistry together with processing-dependent cellular release or contamination. Leukocytes retain nuclei and mitochondria, while platelets retain mitochondria but are anucleate; both are sensitive to cell composition and activation. Erythrocyte, plasma/serum, leukocyte, and platelet measurements should therefore be reported and interpreted separately.

The medication effects remain heterogeneous. A meta-analysis of 20 longitudinal studies found treatment-associated changes in several blood biomarkers, including reductions in whole-blood GPX activity and malondialdehyde in some analyses, but the effects differed by matrix, illness phase, antipsychotic class, and study quality [10]. A separate meta-analysis likewise showed that antipsychotics can modify antioxidant-defence markers without producing a uniform normalising pattern [49]. Antipsychotic exposure should therefore be measured and modelled, not treated as a simple binary explanation.

The GSH-specific evidence is also mixed. A systematic review and meta-analysis by Tsugawa and coworkers found abnormalities in the GSH levels and related enzymes across peripheral and central studies, but with a marked heterogeneity by compartment and clinical context [11]. By contrast, a 2024 proton magnetic resonance spectroscopy meta-analysis found no significant overall medial-prefrontal GSH difference between schizophrenia-spectrum disorders and controls; a small reduction emerged only in the stable-schizophrenia subgroup, and the acquisition sequence, tissue correction, region, medication, and illness phase contributed to heterogeneity [12]. This group-level null is important evidence against a universal central GSH deficit, but it is not equivalent to a high-sensitivity exclusion of smaller, regional, or state-dependent effects. At 3 T, physiological GSH can be unreliably quantified with conventional short-echo PRESS, whereas optimised edited MEGA-PRESS can achieve substantially better precision; sequence choice, voxel placement, spectral overlap, signal-to-noise ratio, and Cramér–Rao lower bounds therefore condition the strength of any null result [50].

Independent cohorts support antioxidant-system abnormalities but do not define one universal enzyme pattern. A broad clinical review emphasised the variation by illness stage, symptom domain, biological matrix, and treatment exposure [51]. In 23 drug-naive first-episode patients and 40 controls, Raffa et al. reported a lower total and reduced GSH, higher GSSG and GPX activity, and lower catalase. In a separate study of 68 patients and 59 controls, erythrocyte catalase and GPX activities were lower in schizophrenia [13,14]. The opposite GPX directions illustrate why isolated enzyme values should not be interpreted as a single disease trait.

Previous work by our group showed that antioxidant defence operates as a coordinated system. The correlation structure among SOD, catalase, GPX, and glutathione reductase differed between symptom-defined male patient groups and controls [52]. In a later cohort comprising 19 first-episode patients, 46 relapsed patients, and 20 controls, the SOD and catalase activities were approximately 40% lower in the first-episode group, whereas the GPX activity was approximately 20% lower in both patient groups. Treatment attenuated SOD and catalase reductions but did not normalise GPX; in the first-episode group, GPX activity correlated inversely with NSS severity [15]. These percentages refer to that single cohort and should not be generalised as pooled effects.

The clinical relevance of NSS is independently supported: meta-analyses show elevated NSS in schizophrenia and, at a smaller magnitude, in unaffected relatives [16,17]. However, the specific inverse GPX-NSS association has not yet been independently replicated. The defensible conclusion is therefore that both antioxidant abnormalities and NSS generalise beyond one laboratory, while their direct biochemical coupling remains provisional.

Longitudinal studies further challenge a simple deficiency model. Drug-naive first-episode cohorts show that risperidone can alter oxidative markers and that baseline antioxidant profiles may be associated with the subsequent clinical response, but the results are marker-specific and not validated for treatment selection [53,54]. Recent first-episode studies have also reported mixed or apparently compensatory patterns, including elevated antioxidant measures alongside oxidative products and associations of GSSG with cognition [55,56]. These findings are compatible with an impaired redox adaptation, not necessarily a uniformly depleted defence.

Taken together, peripheral biomarkers may index systemic vulnerability, compensation, medication effects, inflammatory or metabolic burden, or a state response. They cannot be assumed to mirror a specific brain region. The strongest future designs should integrate peripheral chemistry with central spectroscopy, neurophysiology, cognition, symptom dimensions, and prespecified covariates.

## 5. Genetic and Neurodevelopmental GSH–NMDAR–PV Vulnerability

The developmental redox model places GSH regulation at one possible interface linking genetic liability and environmental oxidative challenges during sensitive periods to PV-interneuron and perineuronal-net vulnerability and impaired oligodendrocyte development. Inadequate GSH synthesis or inducibility may contribute to NMDAR hypofunction and the altered maturation of these systems. These processes could disrupt the gamma synchrony, inhibitory control, critical-period plasticity, long-range connectivity, cognition, and negative symptoms without requiring widespread neuronal loss [3,4,25].

Concrete genetic evidence exists, but it is more circumscribed than a claim that schizophrenia genetics generally converge on redox failure. Variants in the glutamate–cysteine ligase modifier subunit (GCLM) and catalytic subunit (GCLC), which encode the modifier and catalytic subunits of glutamate–cysteine ligase (GCL), have been associated with schizophrenia risk and the reduced inducibility of glutathione (GSH) synthesis in functional cellular studies [18,19]. In early psychosis, a high-risk GCLC genotype was associated with a lower medial prefrontal cortex (mPFC) GSH and altered coupling among the brain glutamate (Glu), brain GSH, and peripheral redox measures [20]. Functional proline dehydrogenase (PRODH) variants also influence proline oxidation and frontostriatal biology, providing an additional route by which amino acid metabolism may intersect with glutamatergic and redox processes [21].

At the polygenic level, an expression quantitative trait locus (eQTL)-informed pathway analysis identified the replicated enrichment of an oxidative stress pathway score in two early psychosis samples [22]. This strengthens the biological plausibility but does not establish a singular redox genetic architecture. The largest genome-wide association study (GWAS) of schizophrenia implicates the broad synaptic and neurodevelopmental biology across many loci [23], and historical candidate genes as a class have not shown a stronger association than the matched noncandidate genes [57]. The calibrated statement is that a subset of genetic liability may alter the redox adaptation, not that genetic liability uniformly converges on GSH failure.

NMDARs are redox-sensitive proteins whose function can be modified through thiol-dependent mechanisms. GSH depletion may reduce NMDAR function directly through oxidative modification or indirectly through calcium dysregulation, mitochondrial stress, inflammatory signalling, and NADPH oxidase (NOX) activation. Conversely, early NMDAR hypofunction can amplify oxidative stress, generating a feed-forward interaction rather than two competing hypotheses [25,58].

PV interneurons are especially vulnerable because sustained fast-spiking activity imposes a high energetic demand. Perineuronal nets provide extracellular-matrix stabilisation and redox protection around many PV cells; oxidative stress during maturation may compromise both the interneuron and its matrix environment, with downstream effects on gamma oscillations and cognition [24,26]. Oligodendrocyte maturation and myelination are likewise influenced by GSH availability, mitochondrial function, and inflammatory signals; experimental and human work links a GSH deficit with impaired myelin maturation and white-matter integrity [27,47].

**Figure 2:**
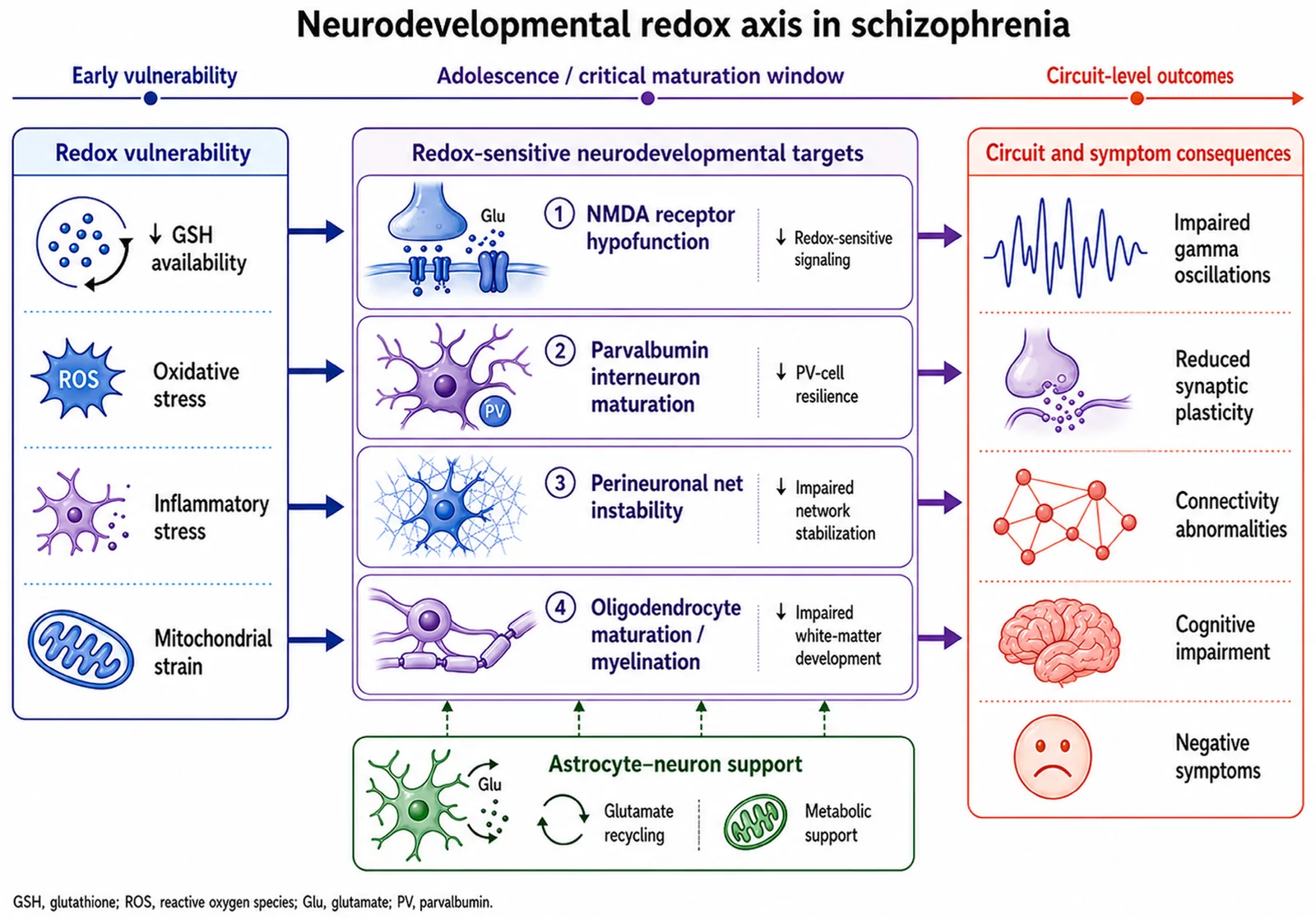
Developmental-window model of redox vulnerability in schizophrenia. The schematic illustrates how reduced GSH availability, oxidative and inflammatory stress, and mitochondrial strain during early vulnerability and adolescent or early-adult circuit maturation may affect NMDAR signalling, PV-interneuron maturation, perineuronal-net stability, oligodendrocyte development, and astrocyte–neuron support. These processes may contribute to altered gamma oscillations, synaptic plasticity, connectivity, cognition, and negative symptoms. The diagram is conceptual and does not establish a proven longitudinal sequence or imply that every patient follows the same pathway. Abbreviations: GSH, glutathione; NMDAR, N-methyl-D-aspartate receptor; PNN, perineuronal net; PV, parvalbumin; ROS, reactive oxygen species.

Figure 2 is deliberately temporal rather than a second version of the broad convergence map. It moves from early vulnerability through the adolescent/critical maturation window to circuit-level outcomes, while illustrating redox-sensitive NMDAR, PV-interneuron, perineuronal-net, oligodendrocyte, and astrocyte-neuron processes. This sequence is conceptual rather than a demonstrated longitudinal trajectory.

## 6. Glutamatergic Homeostasis as a Redox-Linked System

Glutamate occupies a dual position: it is the principal excitatory neurotransmitter and a metabolic precursor for GSH synthesis. Under physiological conditions, presynaptic glutamate is released and cleared predominantly by astrocytic transporters, converted to glutamine, and returned to neurons through the glutamate–glutamine cycle. This arrangement sustains transmission while limiting excitotoxic accumulation [59].

A disturbance at any level may amplify the redox imbalance. Excess extracellular glutamate can promote calcium loading, mitochondrial dysfunction, and reactive-species generation, whereas insufficient NMDAR signalling can impair cortical processing and antioxidant transcriptional responses. The competition for cystine through system Xc- and the altered allocation of glutamate, cysteine, and glycine may simultaneously affect neurotransmission and GSH synthesis.

The glutamate–redox interaction can therefore be organised at three levels: the receptor-level modulation of redox-sensitive NMDARs; the metabolic coupling of amino-acid substrates to GSH synthesis; and the glial control of uptake, recycling, and neuron-astrocyte substrate exchange. This organisation provides a mechanistic bridge without implying that peripheral glutamate or GSH directly reports synaptic function [3,46,59].

## 7. Astroglial, Mitochondrial, and Stress-Reactivity Dynamics

Astrocytes buffer extracellular glutamate, provide metabolic substrates, contribute to GSH homeostasis, regulate inflammatory signalling, and protect neurons from oxidative injury. Single-cell and transcriptomic evidence for coordinated neuron–astrocyte programme changes in schizophrenia supports a systems-level view, although such findings do not by themselves establish redox causality [48].

Mitochondria add a reciprocal layer. Impaired oxidative phosphorylation, calcium buffering, or mitochondrial DNA integrity can increase ROS production, whereas oxidative stress can damage mitochondrial proteins, lipids, and nucleic acids. Human post-mortem transcriptomic and proteomic studies have identified a compromised energy metabolism and oxidative-stress pathways, but the findings differ by region, tissue quality, medication, and agonal factors [28,60].

In an earlier experimental study, Bajic and colleagues introduced a temporal dimension. In astroglial preparations, fluctuating H_2_O_2_ exposure produced stronger mitochondrial membrane hyperpolarisation than continuous exposure, while ATP depletion and increased intracellular calcium occurred across exposure regimes; high-frequency fluctuations also modified NF-kappaB activation [29]. The experiment did not test sleep disruption, relapse, dopamine metabolism, substance exposure, or psychosocial stress. Linking those clinical phenomena to fluctuating redox peaks is therefore a hypothesis for future testing, not a demonstrated extrapolation.

Patient-derived cells provide another level of evidence. An early work reported the abnormal growth of skin fibroblasts from people with schizophrenia [61]. Fournier et al. later applied oxidative challenge and metabolomics to fibroblasts from early-psychosis patients and controls, identifying altered reactivity involving extracellular-matrix or collagen and arginine metabolism; GSH-related and lysolipid responses were particularly altered in carriers of high-risk GCLC genotypes [30]. These peripheral cellular findings support stress-reactivity paradigms but cannot be assumed to reproduce *in vivo* brain pathology.

The clinically relevant construct may therefore be redox resilience, defined here as the magnitude and kinetics of the return to a prespecified homeostatic range after a standardised biological challenge. Candidate operationalisations include a challenge-induced change in GSH or GSH/GSSG (ΔGSH or Δ[GSH/GSSG]), an inducibility ratio such as peak-to-baseline response, a recovery half-time or time to return within 10% of baseline, and the area under the deviation-from-baseline curve. The challenge, biological matrix, sampling schedule, normalisation method, analytical imprecision, and recovery threshold must be specified prospectively. The baseline case–control differences may be modest while repeated or high-demand states reveal impaired inducibility or recovery; longitudinal sampling around acute relapse, treatment initiation, infection, or a standardised *ex*-*vivo* challenge is therefore better suited to this question than one resting measurement [62].

## 8. Neuroimmune Signalling and Redox Dysregulation

Redox and immune signalling are reciprocally coupled. Activated microglia, cytokine signalling, nitric-oxide pathways, and NOX enzymes can increase reactive-species production; reactive species can, in turn, modify the redox-sensitive transcription, inflammasome activity, and cytokine cascades. Nrf2 supports endogenous antioxidant and cytoprotective responses, whereas NF-kappaB coordinates major inflammatory programmes [5,41,63].

A reciprocal network is more plausible than a one-directional chain. NMDAR hypofunction can engage NOX2-dependent oxidative stress and inflammatory mediators, whereas oxidative stress can impair NMDAR function, PV-interneuron maturation, mitochondrial performance, and myelination [4]. Much of the directionality evidence is preclinical; human studies more often demonstrate co-occurrence or association.

This distinction has therapeutic implications. An early-psychosis patient with preserved compensation may differ from a chronic patient with metabolic disease, persistent inflammation, mitochondrial impairment, and negative symptoms. Trials should therefore define the stage and measure the inflammatory as well as antioxidant phenotypes rather than assuming one transdiagnostic redox state.

## 9. Iron–Lipid Redox Regulation and Ferroptosis-Related Mechanisms: A Hypothesis-Generating Extension

Ferroptosis is an iron-dependent form of regulated cell death characterised by the lethal accumulation of phospholipid hydroperoxides when susceptible membrane lipids and pro-oxidant iron overwhelm repair and anti-ferroptotic defences [64]. Its machinery connects iron handling, polyunsaturated phospholipids, GSH availability, and GPX4, making it a biologically coherent extension of a GSH-centred redox model. In this review, iron–lipid redox regulation is the umbrella term for iron handling, phospholipid peroxidation, and the systems that restrain it; ferroptosis-related is reserved for observations involving components of canonical ferroptosis machinery and does not imply cell death. Three evidentiary boundaries apply throughout: ferroptotic neuronal death has not been demonstrated in schizophrenia; plasma immunoreactivity for predominantly intracellular GPX4, ACSL4, Nrf2, and SIRT1 is not a validated circulating concentration without in-matrix validation and source attribution; and the proposed schizophrenia-specific iron–lipid redox loop remains a Tier v, hypothesis-generating inference. Subsequent sections and figures use these definitions.

Human iron findings do not show a simple pattern of excess. Post-mortem work demonstrates a perturbed prefrontal iron biology, whereas quantitative susceptibility mapping and multimodal imaging studies report region- and method-dependent differences that may include lower or redistributed iron rather than uniform accumulation [31,32,33]. A meta-analysis of 39 trace-element studies involving 5151 participants found lower peripheral iron overall across heterogeneous schizophrenia samples [34]. The lower bulk or total iron does not logically exclude a small local redox-active pool if ferritin sequestration, ferroportin-mediated export, or cellular compartmentalisation become uncoupled. However, that reconciliation is a mechanistic possibility, not an observed finding: no schizophrenia study has directly shown that a neuronal labile Fe2+ pool expands while serum or regional bulk iron falls.

Within the canonical pathway, ACSL4 enriches phospholipids with oxidation-prone polyunsaturated fatty acids, system Xc- supports cystine availability for GSH synthesis, and GPX4 reduces lipid hydroperoxides. Ferritin and transferrin influence storage and delivery, while Nrf2 coordinates multiple cytoprotective genes. Under the terminology defined above, conventional iron, ferritin, transferrin, malondialdehyde, and assay-specific GPX4/ACSL4/Nrf2/SIRT1 signals are treated as iron–lipid redox observations rather than evidence of ferroptotic cell death.

The anti-ferroptotic network is broader than GSH–GPX4. FSP1 can regenerate ubiquinol at membranes and suppress lipid peroxidation in parallel with GPX4 [65,66]. Mitochondrial dihydroorotate dehydrogenase provides a CoQ-dependent defence in the inner mitochondrial membrane [67], and the GCH1-BH4 pathway can remodel lipids and provide radical-trapping activity [68]. The thioredoxin–peroxiredoxin system and membrane-repair pathways add further redundancy. Their inclusion prevents the overstatement of a single-axis model.

The plasma measurement of predominantly intracellular or membrane-associated proteins requires explicit validation in the intended matrix. Minimum safeguards include the control of pre-analytical conditions, spike recovery, dilution parallelism, specificity testing against related proteins, orthogonal confirmation, and source attribution; otherwise, the results should be reported as assay-specific immunoreactivity rather than circulating concentration or biological activity. Peng et al. [35] provides one illustrative schizophrenia-specific example whose associations remain hypothesis-generating pending independent, analytically validated replication. Detailed reporting standards are provided in Section 12.

A clinically important competing interpretation is hepcidin-mediated inflammatory iron restriction. A low serum iron and transferrin with high ferritin are characteristic of IL-6/hepcidin-mediated iron sequestration in anaemia of inflammation; elevated malondialdehyde and compensatory antioxidant activity can coexist with chronic inflammatory and metabolic burden [69]. Distinguishing this pattern from an iron–lipid redox phenotype requires haemoglobin and red-cell indices, CRP, IL-6, hepcidin, transferrin saturation, soluble transferrin receptor or the sTfR/log-ferritin index, liver function, preferably longitudinal sampling, and the prespecified control of medication, smoking, diet, nutritional status, metabolic disease, and sample-processing variables.

**Figure 3:**
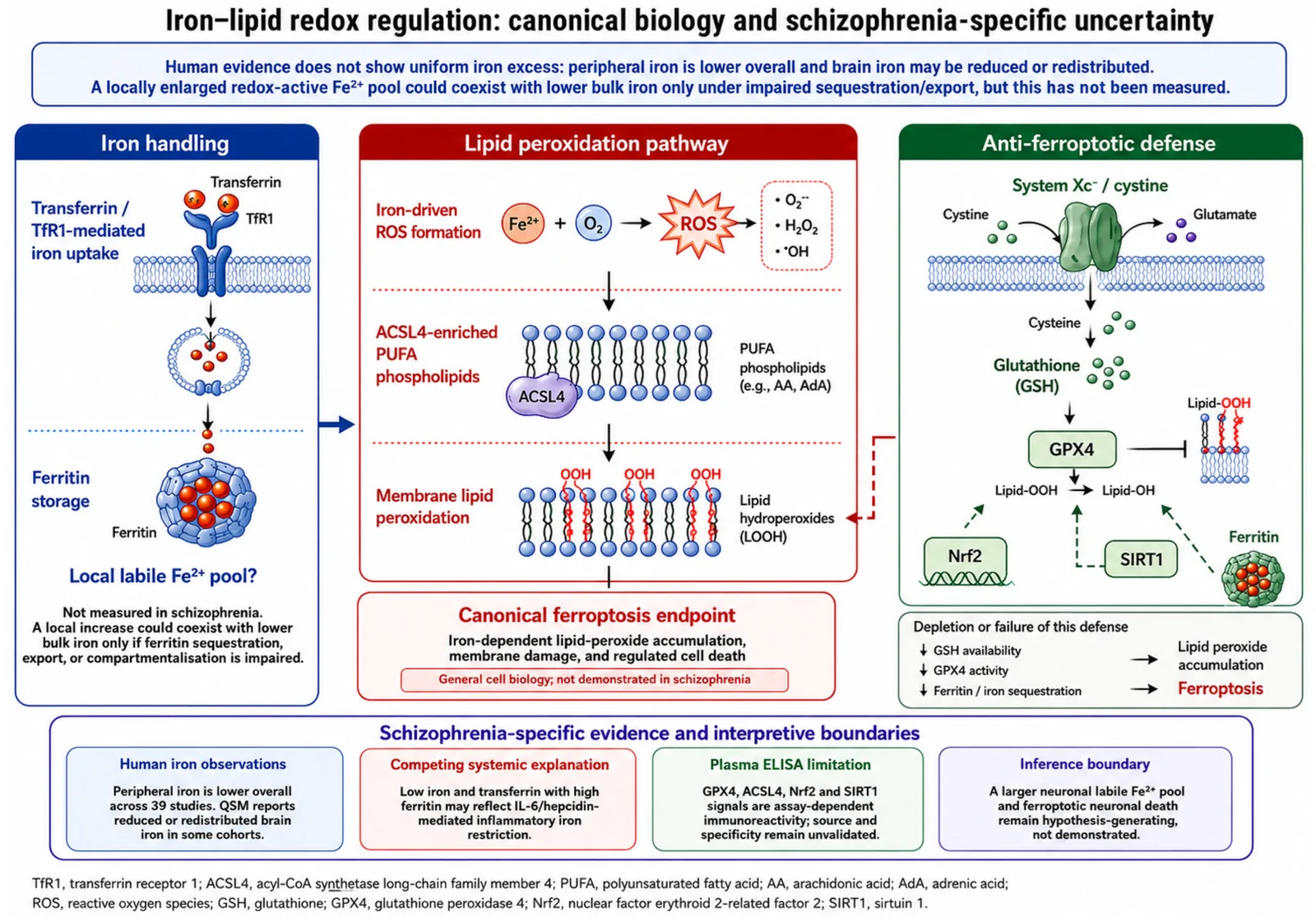
Canonical iron–lipid redox biology and schizophrenia-specific uncertainty. The upper panels depict established cellular pathways involving transferrin-mediated uptake, ferritin storage, ACSL4-enriched phospholipids, system Xc-/GSH/GPX4 defence, and lipid-peroxide accumulation. The lower panel separates human iron observations, hepcidin-mediated inflammatory iron restriction, plasma-assay limitations, and the Tier v inference boundary. The definitions and evidentiary qualifications governing this figure are provided in Section 9. Abbreviations: ACSL4, acyl-CoA synthetase long-chain family member 4; GSH, glutathione; GPX4, glutathione peroxidase 4; Nrf2, nuclear factor erythroid 2-related factor 2; PUFA, polyunsaturated fatty acid; QSM, quantitative susceptibility mapping; SIRT1, sirtuin 1; TfR1, transferrin receptor 1.

Figure 3 separates the established canonical iron–lipid redox biology from the schizophrenia-specific observations and Tier v inference, using the terminology and evidentiary boundaries defined above.

## 10. Integrative Model, Symptom Domains, and Evidence Grading

The evidence can be organised into four interacting loops. The GSH–NMDAR loop links impaired GSH availability or inducibility with redox-sensitive NMDAR function and NOX-related amplification. The mitochondrial–glial loop links bioenergetic and calcium dysregulation with astrocytic support, glutamate clearance, oligodendrocyte maturation, and myelination. The redox–immune loop links inflammatory oxidant production and NF-kappaB signalling with insufficient Nrf2-dependent compensation. The iron–lipid redox loop links altered the iron handling and lipid-peroxide defence with membrane vulnerability, without assuming a systemic or regional total-iron excess. The first three loops have broader human and preclinical support; the fourth remains a hypothesis-generating extension.

Cognitive impairment has plausible connections to redox-sensitive NMDAR plasticity, PV-interneuron gamma synchrony, mitochondrial energy supply, myelin integrity, and membrane lipid homeostasis. Negative symptoms may reflect developmental circuit changes as well as chronic inflammation, metabolic burden, or impaired recovery. These are convergent possibilities rather than established one-to-one mechanisms.

NSSs provide a bedside neurodevelopmental phenotype that is replicated at the group level [16,17]. Their association with GPX in one first-episode cohort is clinically interesting but unreplicated [15]. Similarly, the reported associations between plasma ferroptosis-related markers and cognition or negative symptoms require independent replication and analytical validation [35].

Treatment resistance remains a more tentative endpoint. Persistent redox or inflammatory abnormalities may contribute to incomplete recovery, but no specific redox, GSH, iron, or GPX4 profile has been validated as a mechanism or clinical classifier of antipsychotic resistance. Causal language should therefore be avoided.

## 11. Therapeutic Implications

Redox-directed interventions remain adjunctive and phenotype-dependent. Antipsychotics are central to psychosis management, while candidate adjuncts include GSH precursors, mitochondrial or lipophilic antioxidants, Nrf2-modulating approaches, and anti-inflammatory strategies. None should be presented as a replacement for evidence-based antipsychotic treatment, and none has a validated redox biomarker for routine treatment selection.

N-acetylcysteine (NAC) is the most developed example because it supplies cysteine for GSH synthesis and may influence glutamatergic and inflammatory signalling. A pairwise meta-analysis of eight randomised trials involving 594 participants found no significant improvement in total, positive, negative, or general psychopathology [36]. This result does not support presenting NAC as broadly effective.

A larger network meta-analysis included 49 records representing 50 studies, 2384 participants, and 22 nutraceutical or phytoceutical interventions; the 2384 participants refer to the entire network, not to the NAC node [37]. The NAC estimate was informed by four direct trials plus indirect network evidence and favoured NAC over the placebo for the total symptoms (standardised mean difference −0.87, 95% confidence interval −1.27 to −0.47). This adjunctive effect would exceed the placebo-controlled estimate of most established antipsychotics and approaches the upper end reported across 32 oral agents [38]. Its magnitude should therefore increase, rather than reduce, the concern about sparse-node small-study effects, selective reporting, and publication bias. The heterogeneity was substantial (I-squared 55.9%), the network showed significant global inconsistency (Q = 40.79, 18 degrees of freedom, *p* = 0.002), and the estimate conflicts with the null pairwise NAC meta-analysis [36,37]. It should be treated as an unstable signal of low certainty, not as a competing definitive efficacy estimate.

Ferroptosis-oriented therapeutic strategies remain at the preclinical stage. Iron chelation, radical-trapping antioxidants, lipid-remodelling interventions, and approaches aimed at supporting GPX4 and complementary antioxidant defence pathways have shown promising results primarily in cellular and animal models outside schizophrenia. However, the clinical evidence in schizophrenia is currently lacking, and the systemic manipulation of iron metabolism requires careful evaluation because of the potential adverse effects. Accordingly, these approaches should be regarded as promising research directions rather than established therapeutic options.

GPX4 is a selenoprotein, and selenium utilisation is necessary for its anti-ferroptotic activity in experimental systems [70]. This biology supports measuring the selenium status when interpreting GPX4-related biomarkers; it does not justify empirical selenium supplementation, which carries a narrow safety margin and has not been established for schizophrenia.

The near-term translational objective is therefore the biomarker-informed trial design detailed in Section 12: the stage definition, biological enrichment, prespecified pharmacodynamic targets, and mechanistic outcomes. No circulating, imaging, electrophysiological, or ferroptosis-related marker is currently validated to determine who should receive NAC or another redox-directed intervention.

## 12. Biomarker and Study-Design Strategy

Biomarker proposals are consolidated here to avoid repetition. A research panel could include GSH/GSSG, compartment-appropriate GPX activity, SOD and catalase, malondialdehyde or more specific lipid-peroxidation products, ferritin, serum iron, transferrin saturation, soluble transferrin receptor, hepcidin, CRP, IL-6, selenium, selenoprotein P, and broader nutritional indices. GPX4, ACSL4, Nrf2, or sirtuin 1 should not be treated as routine plasma concentration markers unless analytical specificity and extracellular provenance have first been established. Biochemical measurements should be paired with central or circuit-level measures such as MRS GSH or NAD+/NADH, EEG gamma synchrony, mismatch negativity, cognition, NSS, and structural or functional connectivity. This is a research framework, not a clinically validated panel.

For plasma assays of predominantly intracellular proteins, the minimum analytical reporting should include anticoagulant and tube type, time and temperature before centrifugation, haemolysis/lipaemia/icterus indices, platelet-depleted processing where relevant, storage duration and freeze–thaw cycles, calibration range, duplicate coefficients of variation, spike recovery, dilution parallelism, lot comparison, specificity against homologous proteins (particularly, GPX3 for a GPX4 assay), and orthogonal confirmation by immunoblotting or targeted mass spectrometry. Soluble and extracellular-vesicle fractions should be analysed separately when provenance is part of the hypothesis. Until these criteria are met, results should be reported as assay-specific immunoreactivity rather than absolute circulating protein concentrations.

Covariate control is essential. Studies should prespecify age, sex, gender and hormonal status when feasible, ethnicity or ancestry, body mass index, smoking, diet, physical activity, sleep, alcohol and substance exposure, infection or inflammatory comorbidity, liver and renal function, metabolic syndrome, medication type, dose and duration, illness duration, acute versus stable state, and sample-processing variables. The sex-specific clinical course and antioxidant-treatment associations justify stratification or interaction testing rather than adjustment alone when the sample size permits [71,72]. This is particularly important because the authors’ 2010 antioxidant-enzyme study included only men [52].

Selenium, selenoprotein P, and a broader nutritional status should be measured when GPX4 or selenium-dependent GPX activity is central to the hypothesis. Ferritin must be interpreted alongside complete blood count indices, transferrin saturation, CRP, IL-6, hepcidin, soluble transferrin receptor or the soluble-transferrin-receptor/log-ferritin index, and liver and renal function because ferritin is both an iron-storage and acute-phase protein. Similar care is required for malondialdehyde, which is sensitive to assay chemistry and pre-analytical conditions.

Longitudinal and challenge designs are preferable to isolated case–control comparisons. Repeated sampling around relapse, treatment initiation, remission, infection, or metabolic change can distinguish state effects from persistent vulnerability. A redox-resilience protocol should prespecify the perturbation, matrix, baseline and post-challenge time points, ΔGSH or Δ(GSH/GSSG), inducibility ratio, recovery half-time or return threshold, and area under the deviation curve. *Ex vivo* stress assays and patient-derived cellular models may quantify inducibility and recovery, provided their relation to *in vivo* brain measures is tested rather than assumed.

Study questions should be stage-specific. Clinical-high-risk and first-episode cohorts are suited to transition, symptom emergence, NSS, cognitive trajectory, and early treatment response. Chronic cohorts may be more informative for persistent negative symptoms, cognitive impairment, metabolic burden, and iron–lipid redox patterns. Pooling stages without interaction tests may erase biologically meaningful effects.

Biomarker-enriched trials should prespecify the subgroup rule, analytical measurement range, within-run and between-run precision, clinical endpoint, and evidence that the intervention changed its intended target. Discovery, internal validation, and independent external validation must be separated. For intracellular proteins assayed in plasma, source attribution and orthogonal confirmation should precede any treatment-selection claim. Until those steps are completed, the biomarker-treatment associations remain exploratory.

## 13. Limitations and Future Directions

The evidence base has recurring limitations. Schizophrenia is heterogeneous, and redox markers vary with illness stage, symptoms, sex, smoking, diet, physical activity, sleep, metabolic disease, inflammation, and treatment exposure. Biological matrices are not interchangeable: erythrocytes are anucleate and lack mitochondria, plasma or serum is vulnerable to processing-dependent release and interference, leukocytes retain nuclei and mitochondria, and platelets retain mitochondria but not nuclei. Erythrocyte SOD, catalase, or GPX activity therefore cannot directly report mitochondrial bioenergetics or Nrf2/SIRT1 transcriptional inducibility. Peripheral blood also cannot be assumed to reproduce redox conditions in a specific brain region, while 3 T MRS null findings remain constrained by sequence and regional sensitivity. Many studies are small and cross-sectional, limiting the causal and temporal inference.

Sex is an important limitation in the present literature. Schizophrenia differs by sex in onset, course, symptom expression, endocrine context, medication exposure, and metabolic risk [71]. Male-only or sex-imbalanced samples, including one frequently cited study from the authors’ group, restrict generalisability. Future work should report sex-disaggregated results and test interactions where adequately powered.

Future studies integrating validated analytical approaches with inflammatory iron-status markers and longitudinal designs will be required to distinguish iron–lipid redox alterations from alternative explanations, including inflammation-related iron restriction and clinical or pre-analytical confounding.

Selection and citation bias are additional concerns. Several illustrative findings, particularly the GPX-NSS association and fluctuating-H_2_O_2_ astroglial observations, originate from the authors’ research network and have limited independent replication. Their translational implications are therefore presented as hypotheses, while independent meta-analyses and cohorts are used to contextualise or challenge them.

As a purposive narrative review, the work was not designed to identify every publication or estimate pooled effects. PubMed/MEDLINE was the only database searched systematically, supplemented by citation screening. The exact grouped strings and retained-reference counts are reported in Supplementary Table S1, but the raw database hit counts were not prospectively logged. The study selection was not duplicated, and a formal risk-of-bias or GRADE assessment was not performed. SANRA self-audit improves reporting transparency but does not convert this synthesis into a systematic review, while the certainty labels in Table 1 remain narrative descriptors. Positive mechanistic reports may consequently be overrepresented despite the deliberate addition of null, compensatory, discordant, and competing interpretations.

Future progress requires mechanistic triangulation. Convergence across longitudinal human cohorts, central imaging or neurophysiology, post-mortem tissue, patient-derived cellular models, and experimentally manipulable systems can distinguish correlation from causation. A successful framework should explain heterogeneity and resilience, identify which components are reversible, and show that modifying a defined target improves a prespecified clinical outcome.

## 14. Conclusions

Redox dysregulation remains a coherent framework for integrating neurodevelopmental vulnerability, NMDAR hypofunction, PV-interneuron and perineuronal-net impairment, mitochondrial–glial dysfunction, neuroimmune activation, altered myelination, cognition, negative symptoms, and NSS. The most defensible interpretation is not that oxidative stress alone causes schizophrenia, but that impaired redox adaptation may act as one convergence mechanism whose expression depends on genetic background, illness stage, biological compartment, treatment, and systemic comorbidity.

The hypothesis-generating iron–lipid redox extension adds a testable dimension involving iron handling, phospholipid peroxidation, GPX4, ACSL4, ferritin, transferrin, SIRT1, Nrf2, and parallel FSP1-CoQ, DHODH-CoQ, and GCH1-BH4 defences. Current human evidence supports altered iron handling and selected peripheral associations, but interpretation remains governed by the Section 9 evidentiary boundary.

Progress will depend on longitudinal, sex-aware, multi-compartment, independently replicated studies that separate stage and treatment effects, quantify inflammatory and nutritional covariates, validate assays in the intended matrix, and link peripheral chemistry with central circuit measures. The challenge-based operationalisation of redox resilience may be more informative than resting case–control differences. No redox- or ferroptosis-related biomarker is currently validated to guide treatment selection, and ferroptosis-directed interventions remain experimental.

## Figures and Tables

**Table 1:** Evidence map for the proposed redox convergence framework. Study designs and biological compartments are shown explicitly to prevent equal weighting of replicated human evidence, single-cohort associations, post-mortem findings, and preclinical mechanisms. Tier definitions are provided in Section 2; tiers are assigned to individual principal findings. Certainty terms are narrative, GRADE-informed descriptors rather than formal GRADE ratings.

Domain	Representative Design, Compartment, and Scale	Principal Finding	Evidence Tier(s) and Narrative Certainty	Main Limitation
**Peripheral antioxidant state and trait**	Meta-analysis of 44 studies; meta-analysis of 20 longitudinal treatment studies [9,10]	Antioxidant and lipid-peroxidation measures vary by stage, specimen, and treatment.	Tier i: moderate certainty for stage/matrix dependence, low certainty for any universal signature.	Medication, smoking, diet, inflammation, metabolic status, and sample handling confound interpretation.
**GSH across compartments**	Systematic review/meta-analysis of GSH biology; 2024 MRS meta-analysis [11,12]	Peripheral abnormalities coexist with no overall medial-prefrontal GSH difference.	Tier i: moderate certainty against a universal medial-prefrontal deficit, low certainty for smaller regional or stage-specific deficits.	MRS sequence, voxel, tissue correction, clinical phase, and biological matrix are not interchangeable.
**Antioxidant enzymes and NSS**	Independent cohorts: 23/40 and 68/59; authors cohort: 19 first-episode, 46 relapsed, 20 controls; NSS meta-analyses [13,14,15,16,17]	Enzyme abnormalities and NSS replicate separately; an inverse GPX-NSS association was reported once.	Tier i for the separate phenomena (moderate certainty); Tier ii for their specific coupling (very low certainty).	Small, mainly cross-sectional cohorts; enzyme directions differ; much evidence is erythrocyte-based.
**Genetic GSH vulnerability**	Functional GCLM/GCLC studies, early-psychosis MRS-genotype study, PRODH study, pathway PRS, large GWAS [18,19,20,21,22,23]	A subset of liability may influence redox adaptation.	Tier iii: functional GCLM/GCLC cellular findings. Tier ii: candidate-gene and genotype–MRS associations. Tier i: replicated pathway-score and large-GWAS context. Overall low-to-moderate certainty for a subset effect.	Candidate-gene findings require caution; broad schizophrenia genetics is polygenic and predominantly synaptic/neurodevelopmental.
**Developmental NMDAR–PV–PNN–myelin axis**	Post-mortem meta-analysis, mechanistic reviews, preclinical and translational myelin work [24,25,26,27]	Component findings support a mechanistic bridge from developmental stress to synchrony and connectivity.	Tier i: meta-analytic component findings. Tier iii: post-mortem component observations. Tier iv: mechanistic NMDAR/PNN/myelin evidence. Tier v: the proposed temporal causal sequence. Moderate certainty for component abnormalities; very low certainty for the sequence.	Human longitudinal sequence and individual-level specificity remain uncertain.
**Mitochondrial–glial dynamics**	Human post-mortem omics; in-vitro astroglial exposure study [28,29]	Energy metabolism and stress dynamics may influence redox resilience.	Tier iii: human post-mortem omics. Tier iv: in-vitro astroglial exposure dynamics. Overall low certainty.	Agonal and tissue confounding; exposure-regime and model dependence.
**Stress reactivity**	Patient-derived fibroblast oxidative-challenge metabolomics [30]	Challenge can reveal abnormal metabolic regulation not evident at rest.	Tier iii: very low certainty for brain or clinical generalisation.	Peripheral cellular model; uncertain correspondence to in vivo circuits.
**Iron–lipid redox regulation**	Trace-element meta-analysis; post-mortem and QSM iron studies; cross-sectional plasma study, 204 patients/216 controls [31,32,33,34,35]	Bulk/peripheral iron is often lower; altered handling and assay-dependent plasma immunoreactivity occur in selected cohorts.	Tier i: peripheral trace-element meta-analysis. Tier ii: QSM and cross-sectional plasma associations. Tier iii: post-mortem iron findings. Tier v: an enlarged neuronal labile Fe2+ pool or ferroptotic death. Low certainty for altered iron handling; very low certainty for the mechanistic extension.	No direct labile-iron or ferroptotic-death measure; inflammatory iron restriction and ELISA specificity/provenance are unresolved.
**Therapeutic translation**	Pairwise NAC meta-analysis: 8 RCTs/594 participants; whole nutraceutical network: 50 studies/2384 participants/22 interventions; NAC node informed by four direct trials plus indirect evidence [36,37,38]	Pairwise analysis was null; the network NAC estimate was SMD −0.87 with substantial heterogeneity and global inconsistency.	Tier i: low certainty for adjunctive NAC efficacy, very low certainty for biomarker-selected benefit.	Unusually large sparse-node estimate, inconsistency, indirect comparisons, small-study/publication bias, and no validated selection biomarker.

## Data Availability

No new data were created or analysed in this study. Data sharing is not applicable to this article.

## Supplementary Materials

The following supporting information can be downloaded at: https://www.mdpi.com/article/10.3390/ijms27167105/s1.

## Abbreviations

The following abbreviations are used in this manuscript: ACSL4, acyl-CoA synthetase long-chain family member 4; BH4, tetrahydrobiopterin; CAT, catalase; CoQ, coenzyme Q; CRP, C-reactive protein; DHODH, dihydroorotate dehydrogenase; EDTA, ethylenediaminetetraacetic acid; EEG, electroencephalography; FSP1, ferroptosis suppressor protein 1; GCLC, glutamate-cysteine ligase catalytic subunit; GCLM, glutamate-cysteine ligase modifier subunit; GPX, glutathione peroxidase; GPX4, glutathione peroxidase 4; GSH, glutathione; GSSG, glutathione disulfide; IL-6, interleukin 6; MDA, malondialdehyde; MRS, magnetic resonance spectroscopy; NAC, N-acetylcysteine; NF-kappaB, nuclear factor kappa B; NMDAR, N-methyl-D-aspartate receptor; NOX, NADPH oxidase; Nrf2, nuclear factor erythroid 2-related factor 2; NSS, neurological soft signs; PNN, perineuronal net; PUFA, polyunsaturated fatty acid; PV, parvalbumin; QSM, quantitative susceptibility mapping; RNS, reactive nitrogen species; ROS, reactive oxygen species; SIRT1, sirtuin 1; SOD, superoxide dismutase; sTfR, soluble transferrin receptor; TSAT, transferrin saturation.
