# Supplementary material for: Redox Homeostasis and Oxidative Stress in Schizophrenia: Glutathione–NMDA–Neuroimmune Convergence and a Hypothesis-Generating Iron–Lipid Redox Extension
Source: Int J Mol Sci. 2026 Aug 8;27(16):7105. doi: 10.3390/ijms27167105 (PMC13513858; doi:10.3390/ijms27167105)
Supplement: Supplementary file 1 [file ijms-27-07105-s001.zip › ijms-4442393-supplementary.pdf]

Supplementary Materials

Table S1. Grouped PubMed/MEDLINE search strategy and retained-reference counts

The grouped strings below reconstruct the concept combinations described in Section 2 and are provided to make the purposive narrative-review strategy reproducible.

Search domain	Grouped PubMed/MEDLINE string	Retained references
**Core redox and oxidative stress**	("schizophrenia"[Title/Abstract] OR "psychosis"[Title/Abstract]) AND ("oxidative stress"[Title/Abstract] OR redox[Title/Abstract] OR antioxidant*[Title/Abstract] OR "reactive oxygen species"[Title/Abstract] OR "reactive nitrogen species"[Title/Abstract])	n = 25; refs. 3-6, 10-16, 20-22, 26-30, 33-36, 44, 54, 55
**GSH-NMDAR and neurodevelopmental circuitry**	("schizophrenia"[Title/Abstract] OR "psychosis"[Title/Abstract]) AND (glutathione[Title/Abstract] OR GSH[Title/Abstract] OR GSSG[Title/Abstract] OR "glutathione peroxidase"[Title/Abstract] OR NMDAR[Title/Abstract] OR "NMDA receptor"[Title/Abstract] OR parvalbumin[Title/Abstract] OR "perineuronal net"[Title/Abstract] OR oligodendrocyte*[Title/Abstract] OR myelin[Title/Abstract])	n = 25; refs. 3, 4, 16-18, 23-25, 27-30, 37-48, 53
**Mitochondrial, glial, and neuroimmune biology**	("schizophrenia"[Title/Abstract] OR "psychosis"[Title/Abstract]) AND (mitochondri*[Title/Abstract] OR astrocyte*[Title/Abstract] OR microglia*[Title/Abstract] OR neuroinflamm*[Title/Abstract] OR Nrf2[Title/Abstract] OR "NF-kappa B"[Title/Abstract] OR NOX[Title/Abstract])	n = 16; refs. 4, 5, 11-14, 19, 37, 42, 49-55
**Iron-lipid redox regulation and ferroptosis-related mechanisms**	("schizophrenia"[Title/Abstract] OR "psychosis"[Title/Abstract]) AND (iron[Title/Abstract] OR ferroptosis[Title/Abstract] OR GPX4[Title/Abstract] OR ACSL4[Title/Abstract] OR ferritin[Title/Abstract] OR transferrin[Title/Abstract] OR hepcidin[Title/Abstract] OR "lipid peroxidation"[Title/Abstract] OR phospholipid*[Title/Abstract] OR selenium[Title/Abstract] OR SIRT1[Title/Abstract])	n = 13; refs. 7, 56-66, 70
**Biomarkers, imaging, NSS, and cognition**	("schizophrenia"[Title/Abstract] OR "psychosis"[Title/Abstract]) AND (biomarker*[Title/Abstract] OR plasma[Title/Abstract] OR serum[Title/Abstract] OR erythrocyte*[Title/Abstract] OR "magnetic resonance spectroscopy"[Title/Abstract] OR MRS[Title/Abstract] OR "quantitative susceptibility mapping"[Title/Abstract] OR QSM[Title/Abstract] OR "neurological soft signs"[Title/Abstract] OR cognition[Title/Abstract])	n = 26; refs. 17, 20, 23-36, 40, 49, 53, 57-60, 65, 71, 72
**Genetics and omics**	("schizophrenia"[Title/Abstract] OR "psychosis"[Title/Abstract]) AND (genetic*[Title/Abstract] OR genom*[Title/Abstract] OR GWAS[Title/Abstract] OR GCLC[Title/Abstract] OR GCLM[Title/Abstract] OR PRODH[Title/Abstract] OR transcriptom*[Title/Abstract] OR proteom*[Title/Abstract] OR metabolom*[Title/Abstract])	n = 11; refs. 19, 38-44, 49, 50, 53
**Therapeutic translation**	("schizophrenia"[Title/Abstract] OR "psychosis"[Title/Abstract]) AND ("N-acetylcysteine"[Title/Abstract] OR NAC[Title/Abstract] OR antioxidant*[Title/Abstract] OR selenium[Title/Abstract] OR "iron chelation"[Title/Abstract] OR "radical-trapping"[Title/Abstract] OR nutraceutical*[Title/Abstract]) AND (trial[Title/Abstract] OR treatment[Title/Abstract] OR therap*[Title/Abstract] OR intervention*[Title/Abstract])	n = 10; refs. 5, 22, 33, 34, 67-72

Screening flow: title/abstract relevance screening -> full-text assessment -> prioritisation of systematic reviews/meta-analyses, larger or longitudinal human studies, post-mortem and multimodal investigations, functional genetic studies, and mechanistic reports -> backward and forward citation screening -> evidence-tier assignment and final inclusion. Domain counts are non-exclusive because individual references could contribute to more than one search domain.
